# Alteration of Mevalonate Pathway in Rat Splenic Lymphocytes: Possible Role in Cytokines Secretion Regulated by L-Theanine

**DOI:** 10.1155/2018/1497097

**Published:** 2018-01-15

**Authors:** Chengjian Li, Qiongxian Yan, Shaoxun Tang, Wenjun Xiao, Zhiliang Tan

**Affiliations:** ^1^Key Laboratory of Agro-Ecological Processes in Subtropical Region, National Engineering Laboratory for Pollution Control and Waste Utilization in Livestock and Poultry Production, South-Central Experimental Station of Animal Nutrition and Feed Science in Ministry of Agriculture, Institute of Subtropical Agriculture, The Chinese Academy of Sciences, Changsha, Hunan 410125, China; ^2^College of Pharmaceutical Sciences, Xiangnan University, Chenzhou 423000, China; ^3^Department of Pharmacy, Yongzhou Vocational Technical College, Yongzhou, Hunan 425100, China; ^4^National Research Center of Engineering Technology for Utilization of Botanical Functional Ingredients from Botanicals, Provincial Co-Innovation Center for Utilization of Botanical Function Ingredients, Hunan Agricultural University, Changsha, Hunan 410128, China; ^5^Hunan Co-Innovation Center of Animal Production Safety, CICAPS, Changsha, Hunan 410128, China

## Abstract

L-Theanine is a nonprotein amino acid in tea, and its immunomodulatory function has been confirmed. This study aimed to investigate the effect of L-theanine addition on cytokines secretion in rat splenic lymphocytes and explore its potential immunomodulatory effects on the mevalonate biosynthetic pathway. Our results showed that L-theanine treatment did not influence the proliferation and division indexes of the splenic lymphocytes subsets. Interestingly, L-theanine treatment had regulated the contents of IFN-*γ*, IL-2, IL-4, IL-10, IL-12, and TNF-*α*  (*P* < 0.001) except IL-6 and upregulated the mRNA and protein expression of Ras-related protein Rap-1A (Rap1A), 3-hydroxy-3-methylglutaryl-CoA reductase (HMGCR), and farnesyl diphosphate synthase (FDPs) (*P* < 0.001). Additionally, there was a positive correlation between Rap1A and HMGCR proteins expression and IFN-*γ*, IL-4, and IL-6 levels. In conclusion, L-theanine regulated the secretion of cytokines probably by activating expression of Rap1A and HMGCR proteins involved in the mevalonate biosynthetic pathway in rat splenic lymphocytes. Therefore, L-theanine might be a promising potential drug candidate as immunopotentiator.

## 1. Introduction

Theanine, namely, N-ethyl-*γ*-glutamine, is a unique nonprotein amino acid in tea [1] and has many physiological functions, such as regulating immune response [2, 3], preventing diseases [4], being antitumor [5], relaxing neural tension [6], and antioxidation stress [7]. L-Theanine can be degraded by glutaminase to glutamate and ethylamine* in vivo* [8], and the latter, as a nonpeptide alkylamine antigen, can be subsequently recognized by the gamma delta T-cells (*γδ* T-cells) in peripheral blood; the primed *γδ* T-cells further result in a memory response [3]. The activation of *γδ* T-cells also motivates a nonmemory response to whole bacteria and lipopolysaccharide, which is characterized by IL-12-dependent secretion of interferon-*γ* (IFN-*γ*) and their proliferation. Consequently, the capacity of resistant pathogens can be enhanced [2].

Previous studies have verified that [2, 3, 9, 10] L-theanine can enhance the innate immune function by regulating the secretion of immune cytokines. Wen et al. [11] have proposed that adding 400 mg L-theanine/kg daily in the diets increases the level of secretory IgA in the jejunum and IL-2 and IFN-*γ* in the serum of infant chicken. Bukowski et al. [12] have demonstrated that taking L-theanine in tea beverage into the body of humans induces the innate immune response and immunologic memory. Moreover, clinical study has found that oral administration of L-theanine enhances the activity of *γδ* T-cells, promotes the secretion of IFN-*γ*, and further inhibits tumor activity [12].

The mevalonate biosynthetic pathway is the signal to synthesize terpenoids in eukaryotic cells, and it plays an important role in regulating cell growth, differentiation, and proliferation [13]. It has been reported that the alkylamines (e.g., isobutylamine and sec-butylamine) indirectly stimulate the activation and proliferation of *γ*2*δ*2 T cell by inhibiting farnesyl diphosphate synthase (FDPs) which is a key enzyme in the mevalonate biosynthetic pathway [14] and subsequently results in the accumulation of immediate upstream isopentenyl pyrophosphate (IPP, which can be recognized as an endogenous antigenic components and well-characterized as the first antigen of *γ*2*δ*2 T-cells [15]); then the excessive IPP causes the activation of *γδ* T-cells and promotes the secretion of cytokines consequently [3]. Our recent study has proven that L-theanine administration improves the immunity in the rats by increasing the splenic organ index and developing the status of Th1 drift through decreasing the cytokines ratio of T-helper (Th) cells (IL-4/IFN-*γ*) [16]. However, the mechanism of L-theanine regulated the secretion of cytokines in splenic lymphocytes of rats was unclear. What was the function of mevalonate biosynthetic pathway in the regulation of L-theanine on the immune response of spleen lymphocytes for rats? Therefore, in this study, the differentiation and proliferation, the concentrations of cytokines, the mRNA expression, and protein expression of key enzymes in the mevalonate biosynthetic pathway in rat splenic lymphocytes were assayed. This research will lay the foundation for the development and application of products like immunopotentiator which contain main components of L-theanine.

## 2. Materials and Methods

### 2.1. Chemicals

L-Theanine (Assay: 99.2%, Hongya Yaxing Biotechnology Co., Ltd., Meishan, Sichuan, China); ELISA kits for IL-2, IL-4, IL-6, IL-10, IL-12, IFN-*γ*, and TNF-*α* (Huamei Biotechnology Co., Ltd., Wuhan, Hubei, China); RPMI-1640 medium and fetal bovine serum (FBS, Hyclone, USA); 5-(and-6)-carboxyfluorescein diacetate succinimidyl ester (CFSE) and Rap1A antibody (Santa Cruz, USA); FDPs antibody (Abcam, UK); protease inhibitor cocktail, concanavalin A, and HMGCR antibody (Sigma, Germany); the secondary antibodies (horseradish peroxidase-conjugated goat anti-mouse secondary antibodies, Calbiochem, La Jolla, CA, USA); fluorochrome-conjugated antibodies against rat CD3-PE, CD4-PE-CY7, and CD8-APC antibody and IL-2 (Biolegend, Shanghai, China); Can Get Signal™ Solutions 1 and 2 (Toyobo Co., Osaka, Japan); Super ECL detection kit (KeyGEN Biotech, China); X-ray Kodak film (Eastman Kodak Co., USA); rat splenic lymphocytes separating kit (Haoyang Bio, Tianjin China); phosphate buffered saline solution (PBS, Sigma, Germany) were used; all the other chemicals used in this study were of the analytical purity.

### 2.2. Animals and Management

Animal experiments were conducted according to the animal care guidelines of the Animal Care Committee, Institute of Subtropical Agriculture, the Chinese Academy of Sciences, with an approval number KYNEAAM-2013-0009. All efforts were made to minimize suffering of experimental animals.

Male Sprague-Dawley rats (8 weeks old) employed in this experiments were purchased from Hunan Slack Jingda Laboratory Animal Co., Ltd. (Changsha, Hunan, China). The animals were individually housed in plastic cages on the floor under laboratory conditions (25 ± 3°C, 70 ± 5% relative humidity, good ventilation, and a 12 h light/dark cycle) and had free access to feeds and clean water.

### 2.3. Splenic Lymphocytes Isolation, Cultivation, and Treatment

The method of treatment and anesthesia of the rats was performed according to our recent study [16]; then the spleens from three male rats were quickly collected and washed with PBS solution (pH = 7.4). Fresh spleens were immediately minced in homogenization buffer (10 mL FBS and 15 mL F solution), homogenized by a 2 mL rubber syringe plunger, and filtered through a 74 *μ*m sterile wire mesh nylon screen. The lymphocytes were collected by gradient centrifuging according to the instruction of separating kit. Lymphocytes were resuspended in RPMI-1640 medium, and the erythrocytes were lysed by adding a lysis buffer containing 0.15 M NH_4_Cl, 1 mM KHCO_3_, and 0.1 mM Na_2_EDTA for 5 min at room temperature. Subsequently the lymphocytes were washed twice with 1 mL PBS (pH 7.4) supplemented with 1% BSA and cultured in complete medium which comprised RPMI-1640 medium, 10% FBS, 100 U/mL penicillin, 100 *μ*g/mL streptomycin, and 20 ng/mL IL-2 at 37°C in a 5% CO_2_ humidified environment for 24 h, during which cells were gently vortexed. Then the nonadherent cells (purified lymphocytes) were cautiously collected and cultured for 48 h in the complete medium. Before any treatment, the lymphocytes were resuspended and exhibited >95% viability by trypan blue exclusion.

The doses of L-theanine treatment in this study were 0.25, 1, and 4 mM which were selected according to the results of IFN-*γ* secretion (data not shown). L-Theanine was dissolved in RPMI-1640 medium, filtered through a 0.45-micron filter, and then stored at 4°C before use. When the cells were treated, except the control group (Control), the complete medium in the activation group (without L-theanine treatment) and L-theanine treatments (LTHs) were added with concanavalin A (ConA, 6 *μ*g/mL) to stimulate the proliferation of lymphocytes. All the lymphocytes were separated into five treatment groups: control, an activation group (ConA), and LTHs groups which were separately treated with low lose (0.25 mM), middle lose (1 mM), and high lose (4 mM) of L-theanine for 120 h.

### 2.4. Cytokines Assays

The incubated cells in each treatment were harvested by centrifuging at 400 ×g at 4°C for 5 min. The concentrations of IFN-*γ*, IL-2, IL-4, IL-6, IL-10, IL-12, and TNF-*α* in the supernatant were measured using commercial ELISA kits specific for rats according to the manufacturer's instructions, respectively; the optical densities obtained were normalized to per 2 × 10^4^ cells/well.

### 2.5. CFSE Labeling and Flow Cytometric Analysis

According to the established method [17], the lymphocytes cultured for 48 h were labeled with CFSE. In brief, the cells were pelleted and resuspended in prewarmed (37°C) PBS with 0.1% FBS. Freshly prepared CFSE was immediately added to the cell suspension at a final concentration of 5 *μ*M and the cells were incubated for 10 min at 37°C. Excess CFSE was quenched by adding 10 volumes of ice-cold complete medium and incubating cells for 5 minutes on ice. After staining, the cells were washed twice in RPMI-1640 medium using centrifugation at 400 ×g for 5 min. This staining procedure was optimized so that even highly proliferated daughter cells were easily distinguishable from unlabeled cells during flow cytometric analysis. After the final wash, the cells were separated into five groups (Control, ConA, 0.25 mM L-theanine, 1 mM L-theanine, and 4 mM L-theanine treatments), resuspended with the complete medium, and treated with L-theanine solutions, subsequently transferred to a 96-well plate (Nunclon® Surface, Nunc, Roskilde, Denmark) using 100 *μ*L/well, and then cultivated in a 5% CO_2_ incubator at 37°C for 120 h.

The labeled cells were collected by centrifuging at 400 ×g at 4°C for 5 min and then used to measure percentage of the lymphocytes subsets which expressed CD3, CD4, and CD8 molecules by flow cytometry [18]. Cytometric monoclonal antibodies directed against surface antigens and presented on the lymphocytes containing CD3, CD4, and CD8 were immobilized. In brief, from each subject nine aliquots of 100 *μ*L were prepared: five aliquots corresponding to five groups, respectively, were used for staining, three aliquots were used as control for the antibodies CD3^+^, CD4^+^, and CD8^+^, and one aliquot was used as negative control (no antibody incubated). The staining protocol was performed according to manufacturer's instruction of the antibodies, respectively, and the established method [19]. Aliquots were incubated for 30 min in darkness at 4°C, washed three times with 1 mL of FBS/PBS (400 ×g for 5 min at 4°C), and finally resuspended in 300 *μ*L PBS and kept on ice until analysis.

Cytometric analysis was performed on FACSAria™ II Cell Sorter flow cytometer (Becton, Dickinson Company, USA) in the Central Laboratory of Xiangya Hospital, Central South University, in accordance with the manufacturer's procedure. Acquisition was then performed on a FACSAria™ II Cell Sorter (Becton Dickinson China, China) with BD FACSDiva software (version 6.1.3). CD3^+^CD4^+^CD8^+^, CD3^+^CD4^−^CD8^−^, CD3^+^CD4^+^CD8^−^, CD3^+^CD4^−^CD8^+^, and total lymphocytes were identified by their classical forward scatter and side scatter signals and a minimum of 100,000 lymphocytes from each sample were collected in the gate. Data were analyzed with FlowJo software (version 8.3.2), and the results were finally expressed as percentage of positive cells (%).

### 2.6. Real-Time Quantitative PCR

Primers were designed using the Primer 3 plus program, and sequences are listed in Table 1. The isolation of total RNA, cDNA synthesis, and relative quantification of target genes were performed according to the method of our recent study [16]. All the samples were analyzed in duplicate, and the relative amount of each specific transcript was obtained after normalization against the endogenous control *β*-actin.

### 2.7. Western Blot Analysis

The cells were washed twice with ice-cold PBS and lysed in RIPA buffer (20 mM Tris-HCl, pH 7.5, 150 mM NaCl, 0.1% SDS, 1 mM EDTA, 1 mM EGTA, 1 mM *β*-glycerophosphate, 2.5 mM sodium pyrophosphate, 1% Triton X-100, 1 mM sodium orthovanadate, 10 *μ*g/mL aprotinin, 10 *μ*g/mL leupeptin, and 1 mM PMSF) supplemented with protease inhibitors for 30 min on ice. Lysates were centrifuged at 12,000 rpm for 15 min at 4°C, and the concentration of protein in the supernatant was determined by BCA assay. The electrophoresis, transfer, and blocking of total proteins of cells were the same as the procedures of Yan et al. [20]. The anti-rat primary antibodies (FDPs, 1 : 1000; Rap1A, 1 : 200; HMGCR, 1 : 400; *β*-actin, 1 : 4000) were diluted in Can Get Signal™ Solution 1 and incubated for 24 h at 4°C. After washing with PBS-Tween 20 buffer, membranes were incubated with horseradish peroxidase-conjugated goat anti-mouse secondary antibodies diluted at 1 : 3000 in Can Get Signal™ Solution 2 for 3 h at room temperature. Enzymatic detection of horseradish peroxidase was carried out by a Super ECL detection kit. Densitometric signals were obtained after exposure to an X-ray Kodak film. Band intensities were quantified by Quantity One software (Bio-Rad) and normalized to *β*-actin as internal control for total protein loading. Aliquots of whole cell lysates were subjected to immunoblotting analysis to confirm appropriate expression of proteins.

### 2.8. Statistical Analysis

Statistical analyses were conducted by one-way analysis of variance (ANOVA) using the Mixed Proc of SAS (version 8.2, SAS Institute, Cary, NC, USA). When indicated by ANOVA, means were separated using least significant differences. Duncan's multiple comparison was used to compare differences among different groups. All data are expressed as means ± SD. Significance was declared at *P* < 0.05 or 0.01. The Corr Proc of SAS was used to analyse correlations between relative mRNA abundance of Rap1A, HMGCR, and FDPs genes as well as corresponding proteins expression and the cytokines levels.

## 3. Results

To check whether L-theanine was able to modulate cytokines secretion of active lymphocytes, we firstly determined major cytokines by ELISA. As shown in Table 2, compared with the Control, the levels of IFN-*γ*, IL-2, IL-4, IL-10, IL-12, and TNF-*α* in the ConA and ConA + LTHs groups were increased (*P* < 0.001), therein being the greatest values at 4 mM dose of L-theanine treatment as a whole. ConA and L-theanine treatments resulted in increments in IFN-*γ* and IL-2 production (*P* < 0.001). Compared with the ConA, IFN-*γ* level was elevated (*P* < 0.001) with the increasing doses of L-theanine. Meanwhile, the levels of IL-2, IL-4, IL-6, IL-12, and TNF-*α* in the high dose of L-theanine group were also increased (*P* < 0.001). Additionally, the level of IL-10 was decreased (*P* < 0.001) by ConA and L-theanine treatments.

It is interesting that IFN-*γ*, as a sensitive indication of T-cells activation, was elevated by L-theanine. Whether T-cells activation could further induce proliferation and division was unknown. Hence, we performed a CFSE labeling experiment to obtain the proliferation index (PI) and division index (DI) of the lymphocytes subsets. As shown in Table 3, except that the PI of CD3^+^CD4^+^CD8^+^ cells in 1 mM L-theanine treatment group was greater than that in 0.25 mM L-theanine treatment (*P* < 0.05), all doses of L-theanine treatments did not influence the PI and DI of CD3^+^CD4^+^CD8^+^, CD3^+^CD4^−^CD8^−^, CD3^+^CD4^+^CD8^−^, CD3^+^ CD4^−^CD8^+^, and total lymphocytes.

Due to the failure of proliferation and division of lymphocytes by L-theanine treatment, we carried out real-time quantitative PCR to figure out whether the expression of key genes involved in the mevalonate pathway was regulated by L-theanine. As illustrated in Table 4, compared with the Control, the mRNA expressions of Rap1A (*P* < 0.05) and FDPs (*P* < 0.01) in the ConA + LTHs groups were increased. Compared with the ConA, all doses of L-theanine treatments increased (*P* < 0.01) the mRNA expression of Rap1A and HMGCR; 1 mM L-theanine increased (*P* < 0.05) the mRNA expression of FDPs, but 4 mM L-theanine decreased (*P* < 0.05) its mRNA expression. Moreover, transcripts of all these genes reached a maximum by 1 mM L-theanine treatment.

**Figure 1:**
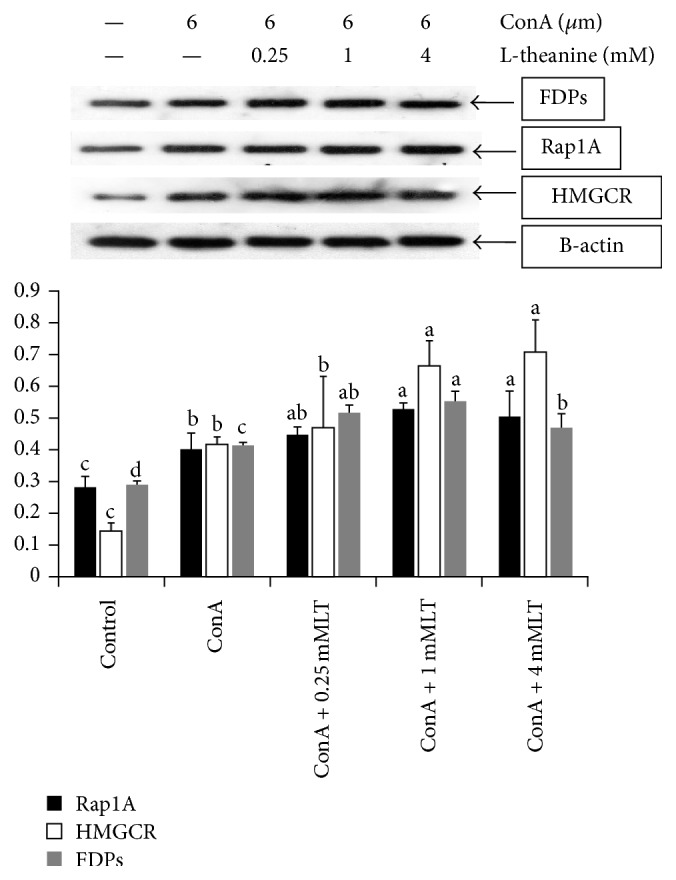
Proteins expression of related genes involved in mevalonate pathway in rat splenic lymphocytes. The cells were treated with 6 *μ*M concanavalin A (ConA), ConA, and different doses of L-theanine (LT) for 120 h; then the proteins expressions of Rap1A, HMGCR, and FDPs were assayed by Western blot. *β*-Actin was used to confirm that equal cell equivalents were loaded. A representative Western blot for each treatment from three independent experiments is shown. Data were expressed as means ± SD (*n* = 4). ^a–d^Means in the same shade not bearing a common letter differ (*P* < 0.05).

Proteins expression of FDPs, Rap1A, and HMGCR in rat splenic lymphocytes treated by L-theanine was shown in Figure 1. Compared with the Control, the abundance of Rap1A, HMGCR, and FDPs proteins in the ConA was increased (*P* < 0.001). Compared with the ConA, the protein expression of Rap1A and HMGCR in the middle and high doses of L-theanine treatments was also upregulated (*P* < 0.001); meanwhile FDPs expression was also increased (*P* < 0.001) by L-theanine treatment, therein being a greatest value at middle dose of L-theanine treatment.

Because the alteration range of Rap1A, HMGCR, and FDP at mRNA level was not in accordance with that at protein level, we did a correlation analysis to figure out which molecular level contributed to the varied cytokines of rat lymphocytes treated by L-theanine. As shown in Table 5, in the lymphocytes treated with L-theanine, there were positive correlations (*P* < 0.01) between the protein expression of Rap1A (*r*^2^ = 0.970), HMGCR (*r*^2^ = 0.985), and IFN-*γ* level. Positive correlations (*P* < 0.05) between the protein expression of HMGCR and IL-4 (*r*^2^ = 0.883) and IL-6 (*r*^2^ = 0.903) levels were also observed. Besides, there were trends of positive correlations (*P* < 0.1) between the protein expression of Rap1A and levels of IL-2, IL-4, and IL-6, between the protein expression of HMGCR and levels of IL-2 and IL-12, and between the protein expression of FDPs and IFN-*γ* level. However, the correlations were not significant (*P* > 0.05) between the mRNA expression and cytokines levels.

## 4. Discussion

### 4.1. L-Theanine Treatment Modulated the Cytokines Secretion of Rat Splenic Lymphocytes

There are about 25% T-cells in splenocytes which secrete cytokines, take part in cell immunity, and regulate the distribution of T-cells subsets in peripheral blood [21]. In healthy status, the ratio of T-helper lymphocyte Th2/Th1 is kept in balance, which is characterized by IL-4/IFN-*γ* for mammalian [22]. Under the impacts of various antigens, cytokines, antigen-presenting cells, and other factors, Th2/Th1 balance might be broken, leading to the balance towards the conversion of Th1 or Th2 status, named Th1 or Th2 drift [23]. As a result, the immune homeostasis of cytokines networks are damaged, causing further changes of immune status as well as the emergence and development of many diseases [24, 25]. Previous studies have shown that L-theanine can enhance the innate immune function by regulating the secretion of immune cytokines [2, 3, 11, 12]. Kamath et al. [2] have reported that delivering 190 mg of L-theanine by human per day enhances the capacity of *γδ* T-cells to secrete IFN-*γ* up to 15-fold in response to challenge with ethylamine or dead bacteria. Kurihara et al. [7] have found that the serum Th2/Th1 ratio can be reduced by L-theanine administration after 6 h and 24 h antigenic stimulation in mice, respectively. Our recent study [16] has shown that L-theanine administration improves the immunity in the rats by increasing the splenic organ index and diminishing the ratio of IL-4/IFN-*γ*. In this study, our results showed that L-theanine treatment promoted the secretion of Th1 cytokines (e.g., IFN-*γ* and IL-2), but decreased IL-4 level and the ratio of IL-4/IFN-*γ*. These data agreed with previous researches and verified again that L-theanine had the capacity of enhancing the body's immune function by regulating the cytokines secretion [2, 3, 9, 10, 12, 26]. Our data showed that L-theanine decreased the expression of IL-4 and IL-6 cytokines after ConA stimulation with 0.25 mM and 1 mM L-theanine treatment whereas it was increased after 4 mM L-theanine treatment. Increments of IL-4 and IL-6 secretion indicated that Th2 subsets were further activated by high dose of L-theanine, consistent with 8 mM L-theanine alone effect (data not shown), and the synergistic effects of ConA and L-theanine dosage might contribute to this result.

### 4.2. L-Theanine Did Not Affect the Proliferation and Differentiation of the Subsets in Splenic Lymphocytes

According to the different molecular phenotypes and functions of the CD, T-cells can be divided into two subsets of CD4^+^ and CD8^+^: the former secretes cytokines to regulate immune response and the latter plays an important role of effector in the stage of immune response. Previous studies have shown that treatment of peripheral blood mononuclear cells from healthy volunteers for 7 days with alkylamines (0.5 mM isopropylamine or sec-butylamine) caused the increase of *γ*2*δ*2 T-cells in the CD3^+^ T population [14]. Bukowski et al. (1995) have noted that *γ*2*δ*2 T-cells have the capacity of recognizing the alkylamines in our foods (e.g., tea, wine, and apple). Moreover, the proliferation and activation of peripheral blood mononuclear cells were induced by alkylamines contained five-carbon atoms treatment. Our data showed that, except the fact that the PI of CD3^+^CD4^+^CD8^+^ cells in middle dose of L-theanine treatment were increased compared with that in lose-dose group, the PI and DI of the total lymphocytes and all of the subsets cells were not influenced by L-theanine. These results implied that L-theanine scarcely influence the proliferation and differentiation of rat splenic lymphocytes which did not agree with the previous findings [12, 14, 26]. The diversity may be due to the difference in the recognization capacity of *γ*2*δ*2 T-cells to alkylamines with various lengths of carbon chains, and there are obvious discrepancies structurally between L-theanine and five-carbon alkylamines. No differences in cell proliferation and division after L-theanine treatment were observed; cholesterol production, ROS generation, and protein farnesylation induced by L-theanine in the rat splenic lymphocytes should be focused in the future.

### 4.3. L-Theanine Activated the Mevalonate Biosynthetic Pathway Which May Play Important Roles in the Secretion of Cytokines in Rat Splenic Lymphocytes

The *γδ* T-cells can be activated by various nonpeptide and stimulatory molecules, therein the first antigen is considered to be IPP which is an intermediate in the mevalonate biosynthetic pathway [15]. It has been reported that nitrogen-containing bisphosphonates, such as zoledronic acid, have the capacity of inhibiting FDPs, consequently causing the accumulation 3 to 5 times of immediate upstream IPP in the target cells (such as osteoclast and all kinds of tumor cells) [27]; then the excessive IPP causes the activation of *γδ* T-cells and promotes the secretion of cytokines consequently [3]. As a result, the tumor cells transform into the activator of *γ*2*δ*2 T-cells [28], the immune cascade reactions are initiated, and the ability of resistance to disease in the bodies is strengthened [29]. Previous study has shown that [14] alkylamines, such as sec-butylamine, have a similar effect on the mevalonate biosynthetic pathway and activation of *γ*2*δ*2 T-cells due to its similar structure to nitrogen-containing bisphosphonates. Thompson et al. (2006) have ever inferred that L-theanine has the similar effect on the mevalonate biosynthetic pathway and activation of *γ*2*δ*2 T-cells like zoledronic acid as well as sec-butylamine. However, our results showed that L-theanine upregulated the mRNA and protein expression of Rap1A, HMGCR, and FDPs in the mevalonate biosynthetic pathway, although the alterations at mRNA level were much greater than that at protein level. The discrepancy may be induced by three causes. Firstly, the low translation efficiency of Rap1A, HMGCR, and FDPs mRNA results in low abundance of protein. Secondly, some posttranslation modifications like Rap1A prenylation, FDPs acetylation, and N-glycosylation and ubiquitination of HMGCR may shorten the half-life period of proteins. Thirdly, the detection sensitivity of real-time PCR and Western blot is inconsistent and the former is more sensitive. Moreover, correlation analysis showed there were positive correlations between FDPs and HMGCR proteins expression and IFN-*γ*, IL-4, and IL-6 contents. These results indicated that the mevalonate biosynthetic signaling was enhanced by L-theanine and might play critical roles in IFN-*γ*, IL-4, and IL-6 secretion in splenic lymphocytes. However, our results showed that the key enzymes abundance was upregulated rather than downregulated as the previous reports in the *γδ* T lymphocytes [3, 14]. Inconsistency between rat splenic lymphocytes response to L-theanine and human peripheral blood mononuclear cells response to the alkylamines [14] occurred due to the different effector cells population. Splenic lymphocytes were complex and composed of B-lymphocytes and a small amount of *γδ* T (CD3^+^CD4^−^CD8^−^) lymphocytes (2%~5%) [3]. Considering our previous finding that the CD3^+^CD4^+^ T lymphocytes in the rat spleen treated with L-theanine alone did not express the proteins involved in the mevalonate biosynthetic pathway but changed cytokines secretion (data was not shown), we inferred that B-lymphocytes or other T subsets may be the targeted cells for L-theanine. Additionally, different recognization capability of lymphocytes to L-theanine and 5-carbon alkylamines may also explain this discrepancy. We inferred that the mevalonate biosynthetic pathway in splenic lymphocytes influenced by L-theanine may result in excessive IPP production which activated the *γδ* T-cells and consequently altered the secretion of cytokines. Further research should be needed to elucidate the function of mevalonate biosynthetic pathway in the process of cytokines secretion and the immunomodulatory mechanisms of L-theanine on other immune cells in spleen.

In summary, L-theanine treatment modulated the secretion of cytokines probably by regulating the mevalonate biosynthetic pathway and consequently enhanced the capacity of immune response to antigen of rat splenocytes. Our study provides a theoretical base for the exploration and application of products as immunopotentiator which contain main component of L-theanine.

## Figures and Tables

**Table 1:** Sequences of primers used for real-time quantitative PCR.

Genes	Accession number	Primer sequence (5′-3′)	Product size (bp)
HMGCR	NM_013134.2	For TCACTGCCATCTACATTG	87
		Rev GACCACTTGCTTCCATTA	
FDPs	NM_031840.1	For CTACAGAGTTCCTATCAG	90
		Rev TTCAGTGTATCTACCAAG	
Rap1A	NM_001005765.1	For TAGTGGTCCTTGGTTCAG	98
		Rev ATCTTCTATCGTTGGGTCATA	
*β*-actin	NM_031144.3	For GGTCAGGTCATCACTATCG	88
		Rev TGCCACAGGATTCCATAC	

**Table 2:** Effects of L-theanine treatment on the cytokines secretion in rat splenic lymphocytes.

Item	Control	ConA	ConA + LTHs			*P* value
			0.25 mM	1 mM	4 mM	
IFN-*γ* (ng/gprotein)	0.24 ± 0.02^e^	0.94 ± 0.04^d^	0.99 ± 0.03^c^	1.25 ± 0.03^b^	1.37 ± 0.05^a^	<0.01
IL-2 (ng/gprotein)	2.60 ± 0.19^d^	5.52 ± 0.16^c^	6.27 ± 0.35^b^	5.46 ± 0.17^c^	7.20 ± 0.26^a^	<0.01
IL-4 (ng/gprotein)	2.45 ± 0.05^e^	29.00 ± 0.2^b^	24.05 ± 0.17^d^	27.83 ± 0.22^c^	31.7 ± 0.31^a^	<0.01
IL-6 (ng/gprotein)	0.21 ± 0.03^b^	0.36 ± 0.04^b^	0.33 ± 0.02^b^	0.35 ± 0.01^b^	0.39 ± 0.02^a^	<0.01
IL-10 (ng/gprotein)	1.70 ± 0.37^d^	3.66 ± 0.38^a^	3.43 ± 0.50^a^	2.51 ± 0.16^c^	3.02 ± 0.28^b^	<0.01
IL-12 (ng/gprotein)	10.24 ± 1.98^d^	44.15 ± 3.2^b^	36.24 ± 1.86^c^	35.00 ± 1.81^c^	52.09 ± 2.45^a^	<0.01
TNF-*α* (ng/gprotein)	2.49 ± 0.19^d^	4.09 ± 0.2^a^	3.52 ± 0.16^b^	3.08 ± 0.23^c^	4.25 ± 0.18^a^	<0.01

^a–e^ Means in the same row not bearing a common superscript letter differ (*P* < 0.05); ConA = concanavalin A activation group; LTHs = L-theanine treatments.

**Table 3:** Effects of L-theanine treatment on the proliferation index (PI) and division index (DI) of the splenic lymphocytes subsets.

Item	Control	ConA	ConA + LTHs			*P* value
			0.25 mM	1 mM	4 mM	
Total lymphocytes PI	2.88 ± 1.58	1.09 ± 0.06	1.77 ± 1.25	1.43 ± 0.59	1.08 ± 0.05	0.191
Total lymphocytes DI	0.04 ± 0.06	0.07 ± 0.01	0.04 ± 0.04	0.14 ± 0.11	0.03 ± 0.05	0.268
CD3^+^CD4^+^CD8^+^PI	2.12 ± 0.05^ab^	2.12 ± 0.03^ab^	1.86 ± 0.44^b^	2.39 ± 0.37^a^	2.1 ± 0.03^ab^	0.306
CD3^+^CD4^+^CD8^+^ DI	0.23 ± 0.17	0.13 ± 0.11	0.30 ± 0.29	0.09 ± 0.04	0.16 ± 0.12	0.685
CD3^+^CD4^−^CD8^−^ PI	2.13 ± 0.97	2.50 ± 1.61	1.11 ± 0.07	2.48 ± 0.48	1.51 ± 0.56	0.378
CD3^+^CD4^−^CD8^−^ DI	0.10 ± 0.08	0.07 ± 0.07	0.49 ± 0.14	0.16 ± 0.17	0.43 ± 0.37	0.093
CD3^+^CD4^+^CD8^−^ PI	2.07 ± 0.03	2.19 ± 0.01	2.13 ± 0.03	2.50 ± 0.63	2.23 ± 0.18	0.481
CD3^+^CD4^+^CD8^−^ DI	0.07 ± 0.01	0.03 ± 0.01	0.15 ± 0.12	0.12 ± 0.09	0.03 ± 0.02	0.242
CD3^+^CD4^−^CD8^+^ PI	2.21 ± 0.22	1.77 ± 0.64	2.22 ± 0.13	1.59 ± 0.77	2.66 ± 1.15	0.511
CD3^+^CD4^−^CD8^+^ DI	0.06 ± 0.05	0.18 ± 0.17	0.06 ± 0.00	0.31 ± 0.25	0.16 ± 0.25	0.581

^a-b^ Means in the same row not bearing a common superscript letter differ (*P* < 0.05); ConA = concanavalin A activation group; LTHs = L-theanine treatments.

**Table 4:** Effects of L-theanine treatment on the mRNA expression of key genes involved in mevalonate pathway in rat splenic lymphocytes.

Item	Control	ConA	ConA + LTHs			*P* value
			0.25 mM	1 mM	4 mM	
Rap1A	1.00 ± 0.05^e^	2.98 ± 0.30^d^	6.23 ± 1.17^b^	11.23 ± 1.51^a^	4.23 ± 0.62^c^	<0.01
HMGCR	1.00 ± 0.11^d^	1.38 ± 0.48^d^	2.65 ± 0.23^b^	4.24 ± 0.77^a^	1.86 ± 0.22^c^	<0.01
FDPs	1.08 ± 0.47^d^	9.82 ± 0.94^b^	9.75 ± 3.03^b^	20.61 ± 1.52^a^	5.53 ± 0.94^c^	<0.01

^a–e^ Means in the same row not bearing a common superscript letter differ (*P* < 0.05); ConA = activation group; LTHs = L-theanine treatments.

**Table 5:** Coefficients between the mRNA and protein expression of FDPs, Rap1A, and HMGCR and the cytokines in rat splenic lymphocytes.

Item	mRNA expression			Protein expression		
	Rap1A	HMGCR	FDPs	Rap1A	HMGCR	FDPs
IFN-*γ*	0.65	0.60	0.60	0.970^*∗∗*^	0.985^*∗∗*^	0.863^#^
IL-2	0.41	0.36	0.35	0.842^#^	0.868^#^	0.768
IL-4	0.51	0.45	0.57	0.869^#^	0.883^*∗*^	0.784
IL-6	0.48	0.42	0.51	0.877^#^	0.903^*∗*^	0.773
IL-10	0.11	0.05	0.25	0.444	0.430	0.496
IL-12	0.28	0.21	0.32	0.763	0.822^#^	0.628
TNF-*α*	−0.03	−0.10	0.05	0.518	0.604	0.376

#, *∗*, and *∗∗* represent *P* < 0.1, * P* < 0.05, and *P* < 0.01, respectively.

## Abbreviations

IPP: Isopentenyl pyrophosphate
Rap1A: Ras-related protein Rap-1A
HMGCR: 3-Hydroxy-3-methylglutaryl-CoA reductase
FDPs: Farnesyl diphosphate synthase
ConA: Concanavalin A
IFN-*γ*: Interferon-*γ*
Control: Control group
LTHs: L-theanine treatments
CFSE: 5-(and-6)-Carboxyfluorescein diacetate succinimidyl ester
ANOVA: Analysis of variance
PI: Proliferation index
DI: Division index.

## Supplementary Materials

Supplementary Materials Highlights of this article.
