# Supplementary material for: Alteration of Mevalonate Pathway in Rat Splenic Lymphocytes: Possible Role in Cytokines Secretion Regulated by L-Theanine
Source: Biomed Res Int. 2018 Jan 15;2018:1497097. doi: 10.1155/2018/1497097 (PMC5820649; doi:10.1155/2018/1497097)
Supplement: Supplementary Materials — Highlights of this article. [file 1497097.f1.doc]

**Highlights**

- L-theanine treatment promoted the secretion of IFN-γ, IL-2, IL-4, IL-10, IL-12, TNF-α and decreased the level of IL-4/IFN-γ in the rat splenocytes.
- It was firstly reported that L-theanine treatment upregulated the mRNA express and protein expression of Rap1-A, HMGCR, FDPs in the mevalonate biosynthetic pathway, and there were positive correlation between these proteins expression and the cytokines secretion.
- The sutdy provided a theoretical base for the exploration and application of products as immunopotentiator which contain main component of L-theanine.
